# Impact of mandatory social isolation measures due to the COVID-19 pandemic on the subjective well-being of Latin American and Caribbean dentists

**DOI:** 10.4317/jced.58776

**Published:** 2022-01-01

**Authors:** María-Claudia Garcés-Elías, Roberto A. León-Manco, Ana Armas-Vega, Andrés Viteri-García, Andrés A. Agudelo-Suárez

**Affiliations:** 1 Faculty of Dentistry. Universidad Peruana Cayetano Heredia. Lima, Peru; 2 Faculty of Dentistry. Central University of Ecuador. Quito, Ecuador; 3 Epistemonikos Foundation, Santiago, Chile; 4 Center for Research in Public Health and Clinical Epidemiology (CISPEC), Faculty of Health Sciences Eugenio Espejo, UTE University, Quito, Ecuador; 5 Faculty of Dentistry, University of Antioquia, Medellin, Colombia

## Abstract

**Background:**

With the spread of the COVID-19 virus, containment measures such as home confinement were implemented, generating stress, anxiety, depression and aggravation of pre-existing diseases in the population, including dentists, who have also been affected due to the risk involved in practicing their profession. Objective: To determine the impact of mandatory social isolation measures on the subjective well-being of Latin American and Caribbean dentists during the community quarantine due to the COVID-19 pandemic in 2020.

**Material and Methods:**

A Cross-sectional study in a sample of 1195 dentists from 21 countries in Latin America and the Caribbean. The main outcome was Subjective Well-Being, evaluated through the World Health Organization Well-Being Index (WHO-5). In addition, sociodemographic characteristics, variables related to the community quarantine due to the COVID-19 pandemic and health variables were considered. A descriptive, bivariate and multivariate (multiple linear regression) analysis was performed to observe the behavior of the variables.

**Results:**

A multiple linear regression analysis was performed, where all the variables included within dimensions, were distributed in a single model, observing an R2% of 9.000 (*p*<0.001), where the R2% change was significant (*p*<0.001) and a constant of 44.190; likewise, within this model, the variable follow-up of preventive measures against COVID-19 reported an unstandardized regression coefficient (b) of 2. 316 (95%CI:1.133-3.499;*p*<0.001), the self-perceived level of concern against COVID-19 obtained a (b) of -5.470 (95%CI:-7.509--3.430; *p*<0.001), the biological sex variable manifested a (b) of -5.417 (95%CI: - 1.157-1.910; *p*<0.001); finally, the level of economic income during compulsory social isolation presented a (b)=5.354 (CI95%:3.461- 7.247; *p*<0.001).

**Conclusions:**

An association was found between subjective well-being and variables related to the social impact of the COVID-19 pandemic, such as following preventive measures, concern about the pandemic and economic factors (decrease in income level), in addition to biologic sex. Follow-up strategies are required for these dental professionals, considering that social isolation measures have continued in many of the countries.

** Key words:**Quarantine, Coronavirus infections, WHO-5, Cross-sectional studies, Latin America, Caribbean Region.

## Introduction

The COVID-19 pandemic imposed challenges for health systems and communities in general, through the drastic and mandatory interruption of the population’s traditional lifestyles, a situation that implied restrictions in mobilization, social interaction and economic activities, resulting in a massive quarantine; consequently, these measures had an alarming impact on the mental well-being of individuals due to the psychological and emotional implications linked to social isolation, which triggered the development of anxiety, depression and post-traumatic stress disorder (1-5).

Likewise, it is important to consider the existence of factors that have a favorable impact on people’s well-being such as sleep quality, eating behavior and physical activity (6). However, the establishment of policies to prevent the spread of COVID-19 such as social distancing and home confinement are opposed to optimal personal well-being and limit individual rights, which would generate stressors that would contribute to generalized emotional distress, the aggravation of pre-existing diseases and the possible emergence of sleep disorders and/or a decrease in the immune response (7-9).

In terms of containment strategies to contain the spread of the disease, compulsory social isolation was adopted by most governments, even though many of these countries were characterized by low or medium incomes and lacked adequate access to basic services, which are essential to comply with the restrictive provisions related to quarantine confinement (1). Likewise, long periods of quarantine, facing economic losses, having a job and the perception of scarce information on the situation from the health authority are closely related to the individual wellbeing perceived in this context (10). In addition to this, the strengthening of remote work stands out as a factor modifying the habits of students and workers; a situation that influenced the loss of job or academic opportunities, salary reduction and student desertion (11).

Similarly, health personnel have been significantly affected in the full exercise of their professional activities due to COVID-19; in the case of dentists, who are also included in this problem, routine dental procedures have been interrupted, limiting it only to emergency and urgent treatments; a situation that denotes the high vulnerability of this occupational group, both because of the danger of contagion and because of financial instability caused by staff cuts and lower demand for care; in addition to the stressors of quarantine. However, scientific evidence at the Latin American level is scarce to describe this situation, despite being a highly relevant problem for dentists, who, like other health professionals, have been affected throughout this pandemic (12,13).

In this sense, the objective of this study was to determine the impact of mandatory social isolation measures on the subjective well-being of Latin American and Caribbean dentists during the community quarantine due to the COVID-19 pandemic in 2020.

## Material and Methods

-Study Setting and Population

This manuscript was written in accordance with the STROBE initiative for observational studies (14). A cross-sectional study was conducted by means of an anonymous survey, provided virtually to a convenience sample of professional dentists and dental students from 21 countries in Latin America and the Caribbean (Argentina, Bolivia, Brazil, Chile, Colombia, Costa Rica, Dominica, Dominican Republic, Ecuador, El Salvador, Grenada, Guatemala, Honduras, Mexico, Nicaragua, Panama, Paraguay, Peru, Puerto Rico, Uruguay and Venezuela), constituting an initial sample of 2442 respondents. We have discarded those surveys with errors in the recording of information, defining a final sample of 2,036 respondents (cooperation rate: 83.4%). It is worth mentioning that, for this study, only the records of general dentists and specialists were considered (n= 1195).

-Data collection techniques

The survey was designed on the Google Forms platform; subsequently, a pilot test was carried out with a sample of 30 participants to evaluate internal consistency and completion time. The time included for the fieldwork was from May 15 to August 26, 2020. Regarding the online questionnaire, it was distributed through digital media such as Facebook groups, WhatsApp messages, e-mails and institutional invitations to different dental schools. The questionnaire collected information about sociodemographic data and incorporated questions about the COVID-19 pandemic (knowledge and practices) and self-perceived well-being. It is worth mentioning that those surveys with errors in the information record were discarded, defining a final sample of 1,195 respondents (cooperation rate: 83.4%).

-Variables

For this research, in addition to the dependent variable, four dimensions were established to group the independent variables present in the study:

Dependent variable: The World Health Organization Well-Being Index (WHO-5) was applied to measure the subjective Well-Being of the participants. This instrument comprises five statements: I have felt cheerful and in a good mood, I have felt calm and relaxed, I have felt active and energetic, I have woken up refreshed and rested and, my daily life has been full of things that interest me; rated on a Likert scale from 0 to 5, where 0=never, 1= occasionally, 2= less than half the time, 3= more than half the time, 4= most of the time, 5= all the time. Subsequently, the scores are added to obtain a total value that must be multiplied by 4, obtaining a Figure from 0 to 100, where the higher the score, the greater the well-being (15).

Socio-demographic characteristics dimension: These include the variables age, biologic sex, Body Mass Index defined as the weight of the individual in kilograms divided by the measurement in meters squared (kg/m2), according to this information is classified as underweight (BMI ≤ 18.50), normal weight (BMI between 18.50 and 24.99), overweight (BMI between 25.00 and 29.99) and obesity (BMI ≥ 30.00) (16). In addition, specialty for those who undertook postgraduate studies, place of origin and level of economic income during compulsory social isolation.

Compliance with mandatory social isolation: This dimension includes variables such as the number of days in mandatory isolation, level of confinement during quarantine and compliance with social distancing.

Dimension of responsibilities during mandatory social isolation: Composed of the number of people with whom he/she lived during quarantine, children under responsibility in quarantine and older adults under responsibility during quarantine.

Preventive behaviors dimension: Comprised by the variables following recommendations to prevent COVID-19 and self-perceived level of concern about COVID-19.

-Statistical Analysis

A descriptive analysis was performed for qualitative and quantitative variables; additionally, the Kolmogorov-Smirnov test was used to evaluate the normality of the distribution of the dependent variable and the other quantitative variables included in the four dimensions. Subsequently, the nonparametric Mann-Whitney U test was applied for dichotomous variables and the Kruskal-Wallis test for polytomous variables. Likewise, a multiple linear regression was developed to generate a model between the independent variables and subjective well-being according to the dimensions of analysis; it should be noted that a logarithmic transformation was previously applied to the WHO-5 due to the lack of a normal distribution. The study had a confidence level of 95%, and in all tests a *p* < 0.05 was considered as an indicator of statistical significance. The SPSS® v. 25.0 program (IBM, NY, US) was used for the analysis.

-Ethics

The present study was approved by the Ethics Committee of the Faculty of Dentistry of the University of Antioquia (Medellin, Colombia; Act 9-2020). Following international guidelines for online surveys, all respondents responded to an informed consent form on the first page of the questionnaire, considering that they had the possibility of declining to participate in the study. Similarly, confidentiality was guaranteed throughout the entire research process, in accordance with the Declaration of Helsinki and the CIOMS guidelines for health research.

## Results

The mean subjective well-being of the sample was 56.83 (SD=18.32); likewise, an association was observed between subjective well-being and the variables age and biological sex (*p*<0.05) (Table 1). In addition, an association was found between subjective well-being and the variables number of days in compulsory isolation, level of economic income in quarantine (*p*<0.05), following preventive measures for COVID-19 and self-perceived level of concern about COVID-19 (Table 2).

**Table 1:**
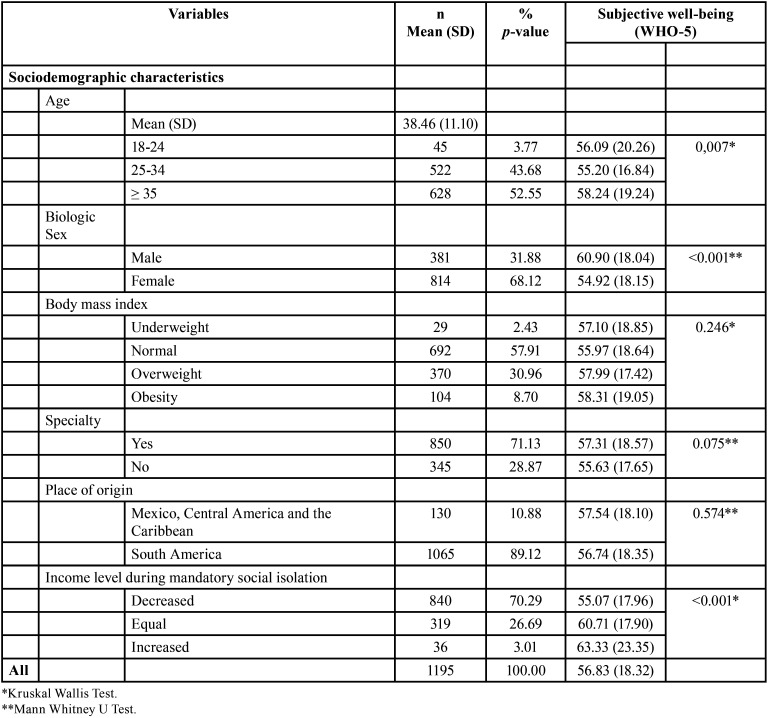
Subjective well-being according to sociodemographic characteristics.

**Table 2:**
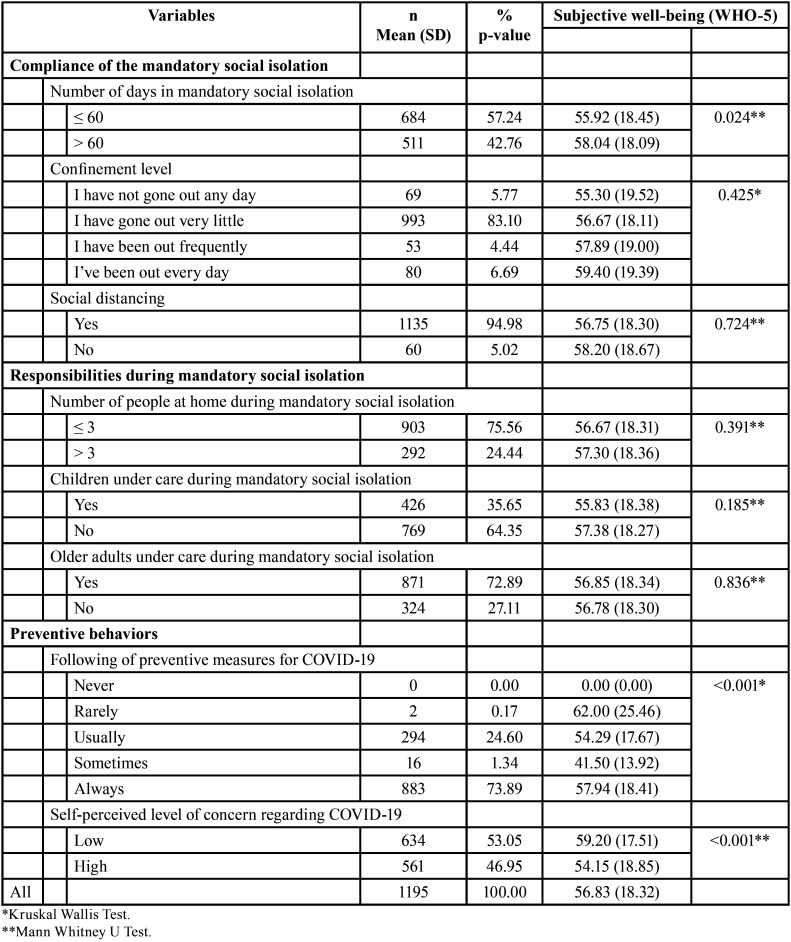
Subjective well-being according to mandatory social isolation.

In addition, a multiple linear regression analysis was performed, where all the variables included within dimensions, were distributed in a single model, observing an R2% of 9.000 (*p*<0.001), where the R2% change was significant (*p*<0.001) and a constant of 44.190; likewise, within this model, the variable follow-up of preventive measures against COVID-19 reported an unstandardized regression coefficient (b) of 2. 316 (95%CI:1.133-3.499; *p*<0.001), the self-perceived level of concern against COVID-19 obtained a (b) of -5.470 (95%CI:-7.509--3.430; *p*<0.001), the biological sex variable manifested a (b) of -5.417 (95%CI: - 1.157-1.910; *p*<0.001); finally, the level of economic income during compulsory social isolation presented a (b)=5.354 (CI95%:3.461- 7.247; *p*<0.001) (Table 3).

**Table 3:**
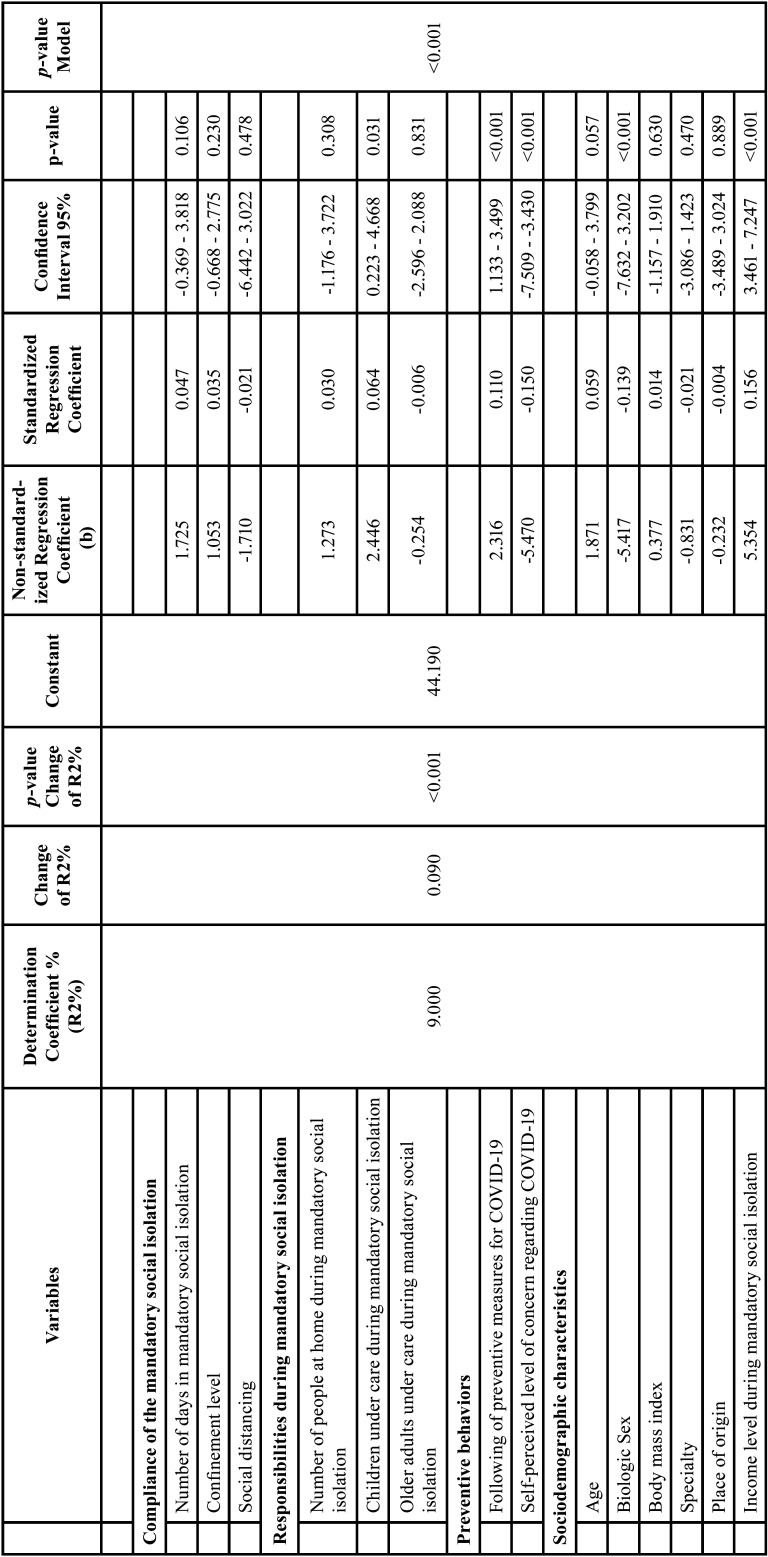
Multiple linear regression for the subjective well-being scores in the study sample (n= 1195).

## Discussion

Due to the high rate of person-to-person transmission of COVID-19, multiple mandatory policies have emerged with the aim of preventing the disease, among them home confinement during quarantine (17). Within this context, people have perceived greater job insecurity in most economic sectors (18), in addition to a reduction in the perception of health and increased feelings of distress (19); which directly affect the individual well-being of the general population, where health personnel are also immersed (1,12).

It is important to point out that according to the findings, this research establishes an explanatory type model, both because of the nature of the study and the model’s capacity to understand how the change of an independent variable affects a dependent variable. In this regard, the study found a negative association between the subjective well-being of Latin American dental professionals and their adherence to COVID-19 preventive measures during community quarantine, which would involve high levels of stress and strain on these professionals to limit opportunities for transmission. Coincidentally, Ahmed *et al*. mention that dentists globally, despite having a high level of knowledge and practices, present a state of anxiety and fear associated with COVID-19 (20); likewise, Putrino *et al*. state that both physicians and dentists are aware of the risk associated with the development of their work activities, in addition to the responsibility that comes with knowing the characteristics of the virus, through accurate and truthful information; as well as the duty to assume a careful and proactive attitude for the protection of their patients and the entire community (21).

On the other hand, because of the high level of concern perceived by dentists due to the high exposure they face to the disease during the development of their professional work, a negative correlation was determined between subjective well-being and the level of self-perceived concern about COVID-19 during community quarantine, which is related to the research done by Mahdee *et al*., where there is a high concern among health care workers, especially dentists, regarding the occurrence, transmission and possibility of contracting COVID-19, coupled with a drastic increase in anxiety; despite the generation of a high level of knowledge on how to deal with the disease by these professionals (22). Likewise, Ahmed *et al*. state that dentists around the world present a state of anxiety and fear, due to the impact that the COVID-19 pandemic has had on humanity (20); as well as that defined by Shacham *et al*. where this disease is associated with high levels of anxiety in dental personnel, due to the fear of contracting it because of having some comorbidity or in those with higher levels of overload (23).

Regarding the existing differences between subjective well-being and biologic sex, studies show that women faced challenging situations during quarantine such as caring for children or older adults and household responsibilities; a situation that was exacerbated in those who worked for the health sector and who presented high levels of concern (20,24).

It is evident that economic stability has great implications on the health and quality of life of people, especially in those professionals who have had to reduce and/or stop their work activities; as can be seen in this study, where there is a positive association between the subjective well-being of Latin American dental professionals and the level of income in the community quarantine during the COVID-19 pandemic, Gasparro *et al*. demonstrated in their study that the interaction between fear of COVID-19 and the perception of job insecurity directly influenced the development of depressive symptoms in Italian dentists during the first quarantine of 2021 (18). Similarly, in the research of Mahdee *et al*. found that, within the COVID-19 pandemic, anxiety among dentists increased drastically, in addition to having seen their income affected with a 50% reduction (22); also, coinciding with Ahmadi *et al*. where they pointed out that dentists faced economic problems due to the closure of dental clinics; likewise, depression and anxiety were common symptoms within this occupational group (25).

In this sense, COVID-19 containment policies, such as home confinement, have a harmful impact on health and can lead to a reduction in the subjective well-being of the general population, as indicated in a recent study applied in 14 countries around the world during the present pandemic. There are certain groups that face a higher risk, such as health professionals (26), for whom following these measures, in addition to other factors, can lead to more psychologically damaging scenarios (27). It is important to mention that the Well-Being Index (WHO-5), an instrument used in this study for the evaluation of subjective well-being, has been applied mostly to the general population (15), resulting in the need to generate more scientific evidence, through this instrument, in health professionals.

Regarding the limitations of this research, the cross-sectional design stands out, which does not allow inferring cause-effect relationships; in addition, there is the possibility of selection bias, since the way of recruiting participants was through e-mails and social networks; similarly, this study included participants from several Latin American countries, where each one has experienced variability in its policies, recommendations, access to information and severity of COVID-19 cases, which could have influenced the response of those who made up the sample and the result of the survey applied. In view of the above, it is suggested that the findings of this research should be interpreted with caution and not generalized.

It is evident that this latest pandemic has generated powerful and drastic changes in the community, affecting both health and individual well-being; it is therefore important to address the implications of the confinement and quarantine by COVID-19 for health professionals, especially dentists, for whom this disease has been challenging in multiple aspects; especially for those living in Latin America, a region where the situation is critical and has been characterized by the collapse of health systems, a dramatic increase in positive cases and containment measures that have not always been effective, in addition to the pre-existing social inequalities that aggravate the development of the disease in those who suffer from it.

## Conclusions

The results of this study show the existence of factors associated with subjective well-being in general dentists and specialists due to confinement by COVID-19. These variables are linked to concern and monitoring of preventive measures against the pandemic, work characteristics (decrease in income level) and biologic sex. Follow-up strategies are required for these dental professionals, considering that social isolation measures have continued in many of the countries and in this sense new studies with quantitative, qualitative and mixed methodologies are required in the Latin American context.lationship quality and mental health during COVID- 19 lockdown.
